# Global prevalence of *Neospora caninum* in rodents: A systematic review and meta‐analysis

**DOI:** 10.1002/vms3.1196

**Published:** 2023-07-07

**Authors:** Yazdan Hamzavi, Yahya Salimi, Mobin Ahmadi, Parvaneh Adimi, Sahab Falahi, Arezoo Bozorgomid

**Affiliations:** ^1^ Department of Medical Parasitology and Mycology School of Medicine, Kermanshah University of Medical Sciences Kermanshah Iran; ^2^ Research Center for Environmental Determinants of Health Health Institute, Kermanshah University of Medical Sciences Kermanshah Iran; ^3^ Student Research Committee Kermanshah University of Medical Sciences Kermanshah Iran; ^4^ Department of Medical Parasitology and Mycology, Faculty of Medicine Tehran Medical Sciences Islamic Azad University Tehran Iran; ^5^ Zoonotic Diseases Research Center Ilam University of Medical Sciences Ilam Iran

**Keywords:** meta‐analysis, Neospora, neosporosis, prevalence, rodent, systematic review

## Abstract

**Background:**

Neosporosis has been considered a cause of abortion in dairy and beef cattle worldwide. Rodents are reservoir hosts for several infectious diseases. It is necessary to determine the prevalence of *Neospora caninum* in rodents to improve the current understanding of the transmission dynamics of *Neospora* as well as its life cycle and risk of transmission to livestock. Therefore, the objective of the present study was to estimate the pooled global prevalence of *N. caninum* in different rodent species.

**Methods:**

Published studies on the prevalence of *N. caninum* in different rodent species were searched in the MEDLINE/PubMed, ScienceDirect, Web of Science, Scopus and Google Scholar and the reference lists of the retrieved articles until July 30, 2022. The eligible studies were selected using inclusion and exclusion criteria. The extracted data were verified and analysed using the random‐effect meta‐analysis.

**Result:**

For this meta‐analysis, a total of 4372 rodents from 26 eligible studies were included. The global prevalence of *N. caninum* in rodents was estimated at 5% (95% CI 2%–9%), with the highest prevalence in Asia (12%; 95% CI 6%–24%) and lowest prevalence in America (3%; 95% CI 1%–14%) and Europe (3%; 95% CI 1%–6%). *N. caninum* was more prevalent in females (4%; 95% CI 2%–9%) than in males (3%; 95% CI 1%–11%). The most common diagnostic test was polymerase chain reaction (PCR) (21 studies). The pooled prevalence of *N. caninum* in rodents based on the diagnostic method was as follows: immunohistochemistry: 11% (95% CI 6%–20%), NAT: 5% (95% CI 4%–7%), IFAT: 5% (95% CI 2%–13%) and PCR: 3% (95% CI 1%–9%).

**Conclusion:**

The results of this study showed a relatively low but widespread prevalence of *N. caninum* infection in rodents.

## Graphical abstract

Graphical Abstract: It is necessary to determine the prevalence of *N. caninum* in rodents to improve the current understanding of the transmission dynamics of *Neospora* as well as its life cycle and risk of transmission to livestock. The results of systematic review and meta‐analysis showed a relatively low (5%) but widespread prevalence of *N. caninum* infection in rodents.

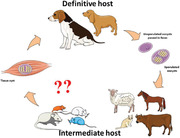

## 1 INTRODUCTION

Neosporosis caused by *Neospora caninum* (Apicomplexa, Sarcocystidae) is an infectious disease of domestic and wild animals worldwide (Nazari et al., 2023). *N. caninum* is similar to another known Apicomplexan parasite called *Toxoplasma gondii*, which is of importance in medicine and veterinary medicine (Nazari et al., 2018). Neosporosis has been considered a cause of abortion in dairy and beef cattle worldwide. This disease can also cause abortion or neonatal mortality in sheep and goats, leading to high financial burdens and enormous economic losses (Benavides et al., 2022).

Domestic dogs (*Canis familiaris*), Australian dingoes (*Canis lupus dingo*), coyotes (*Canis latrans*) and grey wolves (*Canis lupus*) are definitive hosts of *N. caninum* and excrete oocysts in their feces. A wide range of domestic and wild animals as intermediate hosts become infected by ingesting food or water contaminated with sporulated oocysts (Marugan‐Hernandez, 2017). However, host susceptibility to parasite is affected by many factors, such as the type of the host, mode of infection, physiological factors including age, sex, pregnancy and the parasite virulence (Fereig & Nishikawa, 2020).

The consumption of pray (rodents and other small mammals) that could contain *N. caninum* cysts is the main source of infection for definitive hosts. The rodents and other small animals may contribute to the spreading of *N. caninum* infection among domestic or wild dogs through the sylvatic cycle (Almería, 2013). The rodents (order Rodentia) are the largest order of living Mammalia, in the both number of species and the number of individuals. They have a short reproductive cycle and a high compatibility for living in various habitats. In addition, they act as reservoirs, carriers and hosts for several parasitic pathogens (Mohammadi et al., 2022). Various studies have suggested that rodents may play a role in the maintenance of the *N. caninum* life cycle and the dissemination of *N. caninum* in the environment (Benavides et al., 2022; Ferroglio et al., 2003, 2005; Hamidinejat et al., 2011). Although a high prevalence of *N. caninum* has been reported in a number of herbivores and carnivores (Anvari et al., 2020; Ying et al., 2022), limited data are available on the exposure of rodent to *N. caninum*.

Assessment the prevalence of *N. caninum* in rodent is necessary to improve current understanding of the transmission dynamics of *Neospora* and the life cycle and risk of transmission to livestock. Therefore, the objective of this study was to provide tangible evidence on the pooled global prevalence and risk factors of *N. caninum* in different rodent species using previously conducted studies.

## 2 METHODS

### 2.1 Literature review and data sources

Relevant studies on the prevalence of *N. caninum* infection in rodent were searched through the MEDLINE/PubMed, ScienceDirect, Web of Science, Scopus and Google Scholar electronic databases. This meta‐analysis study followed the Preferred Reporting Items for Systematic Reviews and Meta‐Analyses (PRISMA) guidelines (see PRISMA checklist in Additional file 1: Table S1). Two investigators (P.A. and M.A.) searched the relevant studies up to July 30, 2022. The search was limited to studies published in English language.

### 2.2 Search strategy and study selection

A combination of ‘*Neospora*’, ‘*caninum*’, ‘neosporosis’, ‘prevalence’, ‘epidemiology’ and ‘rodent’ was used as search terms. All search results were imported into the Endnote X9 software library, and all duplicates were removed using the same software. Two independent reviewers (A.B. and Y.H.) read the titles and abstracts of all articles retrieved from the databases and excluded those that did not meet the aforementioned selection criteria clearly. Discrepancies were resolved by consultation with a third reviewer (Y.S.). Then, the full‐texts of all potential eligible publications were retrieved and checked by the same reviewers. To identify potential additional data sources, the reference lists of all relevant articles were searched. The following studies were included (1) peer‐reviewed cross‐sectional studies that reported the prevalence of *N. caninum* in rodents, (2) studies with available full‐texts in English language without geographical restrictions (3) and studies published online to July 30, 2022. Studies that (1) compared diagnostic methods, (2) reviews, case reports, (3) papers irrelevant to the topic at issue, (4) studies with a sample size of ≤10 cases, (5) studies including experimentally infected rodents, (6) studies missing information about the total sample size and positive samples and (7) studies with duplicated data were excluded.

### 2.3 Quality assessment

The quality of the included studies was assessed using The Joanna Briggs Institute (JBI) critical appraisal checklist for cross‐sectional/prevalence studies. This checklist evaluates six aspects of a study: sampling process, data analysis process and statistical methods, study settings, measurement tools and response rate. Each item is rated on a scale of 0 (No) to 1 (Yes). The studies that obtained a minimum score of 6 out of 9 were considered high quality and were included in the analysis. Quality assessment was independently done by two reviewers (Y.H. and A.B.). Discussion and consensus were used to resolve disagreements. In this study, all studies were included because their minimum score was 6.

### 2.4 Extraction of data

Data were extracted manually from all relevant studies and recorded in a Microsoft Excel sheet. The extracted variables included authors’ information, publication year, data collection period, animal species, gender, study country and continent, diagnostic method, number of positive cases and sample size. M.A. and S.F. conducted the data extraction from studies, and Y.H. double‐checking the accuracy of the results. Any the discrepancy and inconsistency were settled by discussion, and, if not resolved, a third researcher (A.B.) was consulted.

### 2.5 Statistical analysis

Data were imported to the STATA software version 14 (version 14; Stata Corp). Random‐effects meta‐analysis model using logit transformation was used to estimate the pooled prevalence rates with 95% confidence intervals. Forest plots were used to visualize the heterogeneity among the included studies. The heterogeneity was determined using the *τ* ^2^ and Cochrane *Q* tests. Subgroup analysis was also performed to investigate the overall pooled prevalence of neosporosis in rodents based on the gender, diagnostic method, continent, published year, sample size and tissue type for polymerase chain reaction (PCR). A *p* value less than 0.05 was considered statistically significant.

## 3 RESULTS

### 3.1 Study characteristics

**FIGURE 1:**
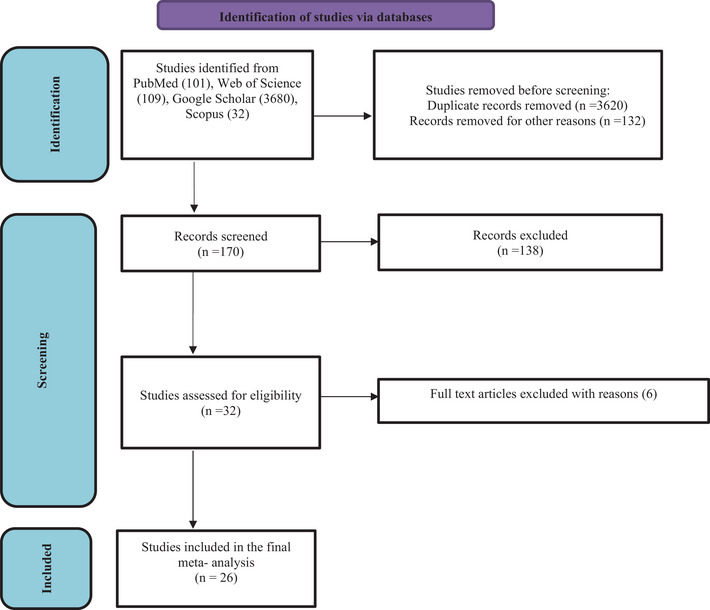
Preferred Reporting Items for Systematic Reviews and Meta‐Analyses (PRISMA) flow diagram describing included/excluded studies.

We identified a total of 3922 records following the initial search of databases; after removing duplicates and/or non‐eligible papers, 26 articles were eligible to be included in this systematic review and meta‐analysis (Figure 1). These 26 studies included 4372 rodents from 15 countries and 5 continents (8 from Europe [1867 rodents], 8 from South America [919 rodents], 7 from Asia [807 rodents], 2 from Oceania [458 rodents] and 1 from North America [321 rodents]). The countries with the highest number of reports were Brazil (six studies) and Iran (four studies). In general, the studies were published between 2004 and 2022. The diagnosis of neosporosis was either performed using serological (IFAT = indirect fluorescent antibody test, 8 studies, ELISA = enzyme‐linked immunoabsorbent assay, 1 study, NAT = *Neospora* agglutination test, 3 studies) and/or molecular (conventional PCR, 11 studies, nested PCR, 6 studies, real‐time PCR, 1 study, heminested PCR, 1 study) and/or histopathology (1 study), immunohistochemistry (2 studies) and/or PAS staining (1 study) and/or bioassay (1 study) and/or parasitology (1 study) methods. The sample type was blood for serological methods and brain, liver, heart, lymph node, spleen, skeletal muscle blood, kidney, muscle and embryo for others methods. The DNA of *N. caninum* was either detected by PCR amplification of ITS1 (1 study), NC5 (13 studies), SAG3 (1 study) or both ITS1 and NC5 (2 studies) loci, in different tissues. All studies were considered eligible for the final meta‐analysis. The references for the included studies and their detailed characteristics are listed in Table 1.

**TABLE 1:** Important characteristics of the included studies (*n* = 26).

Country	Method	Period of sampling	No. samples	No. positive	Species	References
Argentina	IFAT, PCR, histopathology	2014–2015	127	1	*Mus musculus*, *Rattus norvegicus*, *Rattus rattus*	Dellarupe et al. (2019)
Taiwan	IFAT, PCR, immunohistochemistry, PAS staining	–	55	9	*R. norvegicus*	Huang et al. (2004)
Brazil	Agglutination test	–	166	8	*R. rattus*, *Kerodon rupestris*	Lima et al. (2019)
Australia	Nested PCR	–	104	28	*Mus domesticus*	Barratt et al. (2008)
Thailand	PCR	2011–2016	192	43	*Bandicota*, *Berylmys*, *Maxomys*, *Menetes*, *Mus*, *Niviventer*, *Rattus*, *Hylomys suillus* ^a^, *Suncus murinus* ^a^, *Tupaia glis* ^a^	Japa et al. (2018)
USA	Agglutination test, Nested PCR, Bioassay	1999–2005	321	15	*R. norvegicus*, *M. musculus*	Jenkins et al. (2007)
Spain	PCR	2012–2013	313	4	*Apodemus sylvaticus*, *M. musculus*, *Mus spretus*, *R. rattus*, *Crocidura russula* ^a^	Fernández‐Escobar et al. (2020)
Iran	PCR	2015	70	0	*Meriones persicus*, *M. musculus*, *Cricetulus migratorius*	Nazari et al. (2019)
The Netherlands	Real‐time PCR	2004	250	31	*M. musculus*, *Microtus arvalis*, *A. sylvaticus*, *R. norvegicus*, *Micromys minutus*, *Myodes glareolus*, *Microtus agrestis*, *Sorex araneus* ^a^, *C. russula* ^a^	Meerburg et al. (2012)
UK	Nested PCR	2004	145	5	*M. domesticus*, *R. norvegicus*	Hughes et al. (2006)
Brazil	PCR	–	26	6	*Hydrochaeris hydrochaeris*	Truppel et al. (2010)
UK	Nested PCR	–	248	8	*A. sylvaticus*	Thomasson et al. (2011)
Brazil	IFA	2003	213	20	*H. hydrochaeris*	Yai et al. (2008)
Italy	PCR	2011–2012	78	0	*R. rattus*	Zanet et al. (2014)
Mexico	Nested PCR, IHC	2011	33	23	*M. musculus*, *R. norvegicus* and rock squirrels (*Spermophilus variegatus*)	Medina‐Esparza et al. (2013)
Czech Republic–German	PCR	2006–2009	360	13	*M. musculus*, *M. domesticus*	Hůrková‐Hofmannová et al. (2014)
Iran	IFAT	2015	157	32	*M. persicus*, *M. musculus*, *C. migratorius*	Nazari et al. (2020)
Brazil	Heminested PCR	2005–2008	121	0	*R. rattus*, *R. norvegicus*, *M. musculus*	Muradian et al. (2012)
Iran	Agglutination tests (NAT)	2016–2017	150	9	*R. rattus*	Mosallanejad et al. (2018)
Austria	PCR	2004	354	6	*M. arvalis*, *Arvicola terrestris*	Fuehrer et al. (2010)
Brazil	IFAT		63	2	*Hydrochoerus hydrochaeris*	Valadas et al. (2010)
Brazil	IFAT	2012–2013	170	0	*H. hydrochaeris*	de Abreu et al. (2016)
Italy	PCR	–	233	25	*M. musculus*, *R. norvegicus*, *A. sylvaticus*	Ferroglio et al. (2007)
Iran	Nested‐PCR	2017	71	23		Gharekhani et al. (2022)
Czech Republic	PCR, cELISA	2002, 2011–2014	240	1	*Apodemus flavicollis*, *A. sylvaticus*, *Apodemus agrarius*, *M. glareolus*, *M. arvalis*, *Microtus subterraneus*, *M. musculus*, *Crocidura suaveolens* ^a^, *S. araneus* ^a^	Machačová et al. (2016)
India	IFAT, Parasitological examination of tissues	2014–2015	112	12	*R. rattus*	Dhandapani et al. (2017)

Abbreviations: IHC, immunohistochemistry; PCR, polymerase chain reaction.

^a^It is not a rodent.

### 3.2 Results of meta‐analysis

**FIGURE 2:**
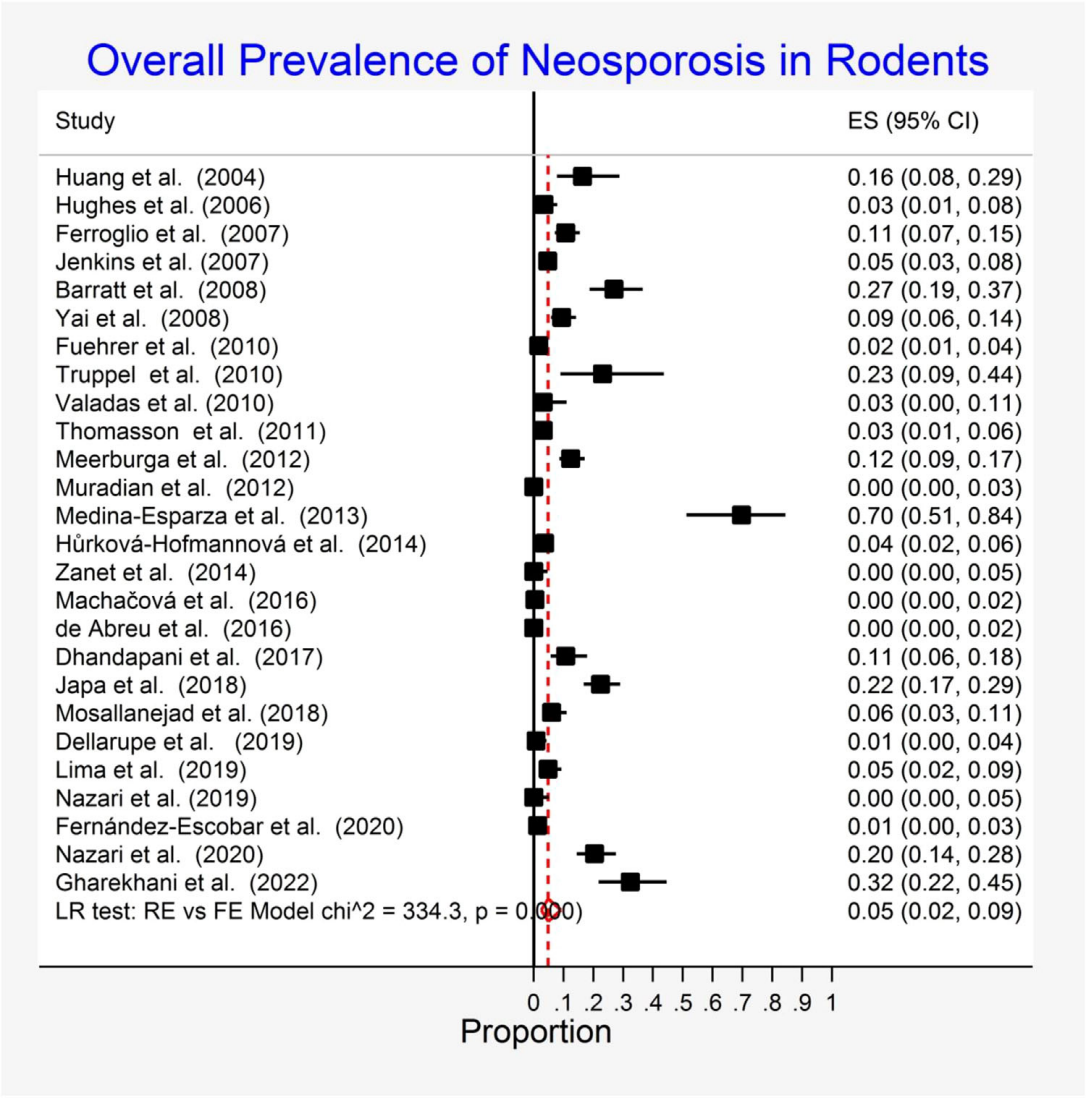
Forest plot of the global prevalence of *Neospora caninum* in rodents.

The pooled prevalence of *N. caninum* was estimated at 5% (95% confidence interval [CI]: 2%–9%) in rodents worldwide (Figure 2). The included studies demonstrated a strong heterogeneity (*Q* = 334.3, df = 24, *p* < 0.001, *τ* ^2^ = 2.89). Sub‐group analysis was performed based on gender, sample size, published year, continent, diagnostic method and tissue type for PCR (Table 2).

**TABLE 2:** Pooled prevalence of *Neospora caninum* in rodents in different subgroups.

Subgroup variable	No. of studies	Prevalence (95% CI)	*τ* ^2^	Heterogeneity (*Q*)	df	*p* Value
**Year**						
<2011	9	8 (4–14)	4.68	282.75	15	<0.001
≥2011	17	3 (1–9)	0.87	57.34	7	<0.001
**Gender**						
Male	5	3 (1–11)	1.31	15.64	2	<0.001
Female	5	4 (2–9)	0.34	1.89	3	0.08
**Sample size**						
≤150	12	5 (1–16)	5.30	166.21	10	<0.001
>150	14	5 (2–9)	1.42	150.93	12	<0.001
**Continent**						
America	10	3 (1–14)	5.24	115.43	7	<0.001
Asia	7	12 (6–24)	0.98	33.29	5	<0.001
Europe	8	3 (1–6)	2.89	49.30	6	<0.001
Oceania	2	7 (5–10)	0.00	–	1	–
**Diagnostic method** ^a^						
IFAT	8	5 (2–13)	1.75	46.87	6	<0.001
NAT	3	5 (4–7)	0.0	0.0	1	–
PCR	21	3 (1–9)	6.44	491.50	19	<0.001
Immunohistochemistry	2	11 (6–20)	–	–	–	–
**Tissue type for molecular diagnosis** ^b^						
Brain	15	2 (1–6)	4.24	328.56	13	<0.001
Heart	4	2 (0–13)	3.19	21.42	2	<0.001
Skeletal muscle	2	6 (4–9)	–	–	–	–
Liver	4	5 (0–36)	6.67	99.72	2	<0.001

Abbreviations: CI, confidence interval; PCR, polymerase chain reaction.

^a^ The prevalence of *N. caninum* using other diagnostic methods, such as PAS staining, histopathology, bioassay and parasitology was zero.

^b^ The number of studies examining *N. caninum* in blood, spleen, kidney and lymph nodes was <2.

In a subgroup analysis based on continent, the highest pooled seroprevalence of N. caninum in rodents was seen in Asia (12%; 95% CI 6%–24%), followed by Oceania (7%; 95% CI 5%–10%), America (3%; 95% CI: 1%–14%) and Europe (3%; 95% CI: 1%–6%). The pooled prevalence of *N. caninum* was higher in females (4%; 95% CI 2%–9%) as compared to males (3%; 95% CI 1%–11%). The findings showed that studies published in 2011 or after reported a higher prevalence of 5% (95% CI 2%–9%) for *N. caninum* vs. 3% (95% CI 1%–9%) in studies conducted before 2011. The overall prevalence of *N. caninum* in rodents based on the sample size was 5% (95% CI 1%–16%) in the sample size ≤150 and 5% (95% CI 2%–9%) in the sample size >150.

The PCR method was the most common diagnostic test to detect *N. caninum* (21 studies). The pooled prevalence of *N. caninum* in rodents based on the diagnostic method was as follows: immunohistochemistry: 11% (95% CI 6%–20%), NAT: 5% (95% CI 4%–7%), IFAT: 5% (95% CI: 2%–13%) and PCR: 3% (95% CI 1%–9%). The subgroup analysis details are showed in Table 2.

## 4 DISCUSSION

Rodents can play an important epidemiological role in the spread of neosporosis because they are preyed by canids, which in turn shed resistant oocysts into the environment through their feces and spread the infection. In addition, they are found in almost every terrestrial habitat and can easily move between habitats and possibly spread the infection within different communities. Considering the veterinary and economic importance of neosporosis, we conducted the first systematic review and meta‐analysis to evaluate the prevalence of *N. caninum* in rodents worldwide.

Based on the results of the present meta‐analysis, 5% (95% CI 2%–9%) of rodents are infected with *N. caninum* across the world. This prevalence rate is lower than *N. caninum* seroprevalence in cattle (23%; 95% CI 19%–27%) (Ansari‐Lari, 2021), domestic cats and wild felids (15%; 95% CI 10%–21%) (Nazari et al., 2023) and sheep (13%; 95% CI 10%–15%) (Morales et al., 2022). A possible explanation for the low prevalence found is that the most common diagnostic method used in the studies included in the present study was molecular tests. The molecular technique is rarely used in larger living mammals due to the ethical limitations of parasite isolation. The PCR Neospora suffers from lack of standardization and variable performance according to the laboratory. (Akya et al., 2020). Therefore, the overall estimate may not be a true estimate of the infection rate in rodents and should be interpreted with caution due to this unavoidable limitation.

The findings of this systematic review and meta‐analysis indicated that Asia (12%) and Oceania (7%) had the highest prevalence estimates compared to America and Europe (3% both). Nonetheless, attention should be paid to the small number of studies from Oceania (two studies) and lack of any reports from Africa. A high prevalence of *N. caninum* in intermediate hosts is assumed to be associated with the presence of dogs. *N. caninum* only occurs where canids are present. Zanet et al. (2014) investigated the epidemiology of *N. caninum* in *Rattus rattus* in the absence of domestic reservoirs and definitive hosts in Montecristo Island (Italy) and reported no evidence of the presence of *N. caninum* (Zanet et al., 2014). It should be noted that *N. caninum* can maintain its life cycle for an indeterminate duration without the involvement of the definitive host through endogenous transplacental transmission (from persistently infected animals) (González‐Warleta et al., 2018).

Different techniques were used for the detection of *N. caninum* in rodents (e.g. parasitology, histology, serology, immunochemistry and conventional and real‐time PCR). The prevalence estimate obtained with immunohistochemistry (11%) was higher than IFAT (5%) as the gold standard test for neosporosis and NAT (5%) and PCR techniques (3%). PCR was the most common test for the diagnosis of *N. caninum* (21 studies). With respect to the applicability of PCR for the diagnosis of cyst‐forming parasites in tissue specimens, it is worth noting that this approach has intrinsic difficulties for the diagnosis of this type of infection; for example, small amount of the parasite in the tissue, the target DNA and primers and the organ of choice for detection of infection can influence the outcome of PCR (Rostamian et al., 2021). Thus, PCR results for the detection of cyst‐forming coccidia in tissue samples should be interpreted with caution because of false negative results.

Many PCR‐based methods have been developed to target different genes or gene regions specific to *N. caninum* such as SAG3, 18S rDNA, 28S rDNA, ITS1 and Nc‐5. The most popular gene target is the Nc‐5 gene because it is highly specific and not found in the genome of Toxoplasmatinae subfamily, such as *T. gondii*, *Hammondia* spp., *Sarcocystis* spp. and *Besnoitia besnoiti* (Correa‐Castro et al., 2021; Mamaghani et al., 2023; Yamage et al., 1996). Overall, in present systematic review and meta‐analysis, NC‐5 was used for detection of *N. caninum* DNA in 13 molecular studies. Beside, *N. caninum* DNA detection was investigated in different tissues, including the blood, liver, spleen, kidney, lymph node, brain, heart or skeletal muscle, whereas the parasite DNA was identified more frequently in the skeletal muscle sample (6%) compared to the brain, liver and heart tissue samples from the same animals (2%, 5%, 2%, respectively). On the contrary, some researchers believe that protozoan cysts and tachyzoites are most likely to be found in the brain and spinal cord, and intracellular tachyzoites can also be detected in myocytes and myocardial Purkinje fibres (Donahoe et al., 2015; Nishimura et al., 2013). However, it should be noted that in the present systematic review, the detection of the parasite DNA in the skeletal muscle was investigated in only two studies; therefore, the results should be interpreted with caution.

A key strength of this systematic review and meta‐analysis is that it determined the pooled prevalence estimates of *N. caninum* among rodents across the world for the first time. Nevertheless, this meta‐analysis had several limitations, including a small number of studies and also uneven distribution of eligible studies across different continents, lack of information about risk factors, and variations in diagnostic methods with different sensitivity and specificity indexes. Overall, the estimated prevalence rates should be interpreted considering these limitations.

## 5 CONCLUSION

The results of this study showed a relatively low but widespread prevalence of *N. caninum* infection in rodents. The findings of the present study provide a better picture of the epidemiology of *Neospora* among rodents and may be used to improve the existing knowledge of the role of these small mammals as intermediate hosts in the life cycle of *Neospora*.

## AUTHOR CONTRIBUTIONS

Arezoo Bozorgomid and Yazdan Hamzavi designed the study. Mobin Ahmadi, Parvaneh Adimi, Sahab Falahi and Yazdan Hamzavi collected the data. Yahya Salimi conducted the statistical analyses. Arezoo Bozorgomid and Yazdan Hamzavi drafted the first version of the paper. All co‐authors made a substantial contribution to interpreting data, critically revised the article and approved the final version, including the authorship list.

## CONFLICT OF INTEREST STATEMENT

All the authors declare no conflicts of interest.

## ETHICS STATEMENT

The protocol was approved by the Ethics Committee of Kermanshah University of Medical Sciences (IR.KUMS.MED.REC.1401.268).

### PEER REVIEW

The peer review history for this article is available at https://publons.com/publon/10.1002/vms3.1196

## Supporting information

Supporting Information

## Data Availability

All data generated or analysed during this study are included in this published article.
